# Elegant Prescriptions

**Published:** 1912-10-19

**Authors:** 


*To the Editor of* The Hospital.

### Elegant Prescriptions.

*To the Editor of* The Hospital.

Sir,—I venture to submit an improvement on Dr. B. Bramwell's hydrarg. perchlor. and iodide mixture:

℞ Liq. hydrarg. perchlor. ... ʒss.

Calc. iodidi ... ... ... gr. v.

Aq. menth. pip. ad ... ... ℥ss.

Ft. mist. ℥viij.

This mixture has no objectionable taste, and has the advantage of the presence of calcium, a salt that is of value in syphilis.—Yours faithfully,

G. Arbour Stephens.

P.S.—Another "elegant" preparation is:

℞ Liq. ferri perchlor. ... ... ♏x.

Calc. chloridi ... ... ... gr. x.

Glycerole pepsin ... ... ♏xv.

Glycerin. pur. ... ... ʒj.

Aq. ad ... ... ... ... ℥j.

Ft. mist. ℥viij. ℥ss. t.d.s. p.c.

